# Understanding Gram-Negative Bacterial Infections in Thailand: An Analysis of Trends and Challenges

**DOI:** 10.4269/ajtmh.25-0170

**Published:** 2025-12-02

**Authors:** Sydney F. Denney, Narisara Chantratita, Paul J. Brett, Mary N. Burtnick

**Affiliations:** ^1^ Department of Microbiology and Immunology, University of Nevada, Reno School of Medicine, Reno, Nevada;; ^2^ Department of Microbiology and Immunology, Faculty of Tropical Medicine, Mahidol University, Bangkok, Thailand;; ^3^ Mahidol-Oxford Tropical Medicine Research Unit, Faculty of Tropical Medicine, Mahidol University, Bangkok, Thailand

## Abstract

Gram-negative bacteria pose a significant threat in hospitals and community settings across Thailand. Limited antimicrobial stewardship, access to and use of prevention measures, and gaps in national surveillance contribute to this ongoing global challenge. In the present review, the literature on Gram-negative hospital- and community-acquired infections in Thai adults published between 2010 and 2024 is summarized, focusing on data collection and reporting gaps. *Acinetobacter baumannii*, *Klebsiella pneumoniae*, *Escherichia coli*, *Pseudomonas aeruginosa*, and *Burkholderia pseudomallei* were the most frequently reported pathogens. Of these, *A. baumannii, K. pneumoniae*, and *P. aeruginosa* were the most common hospital-acquired infections, whereas *E. coli* and *B. pseudomallei* were the most common community-acquired infections. Although there is a critical need for studies on antibiotic resistance patterns, treatments, and specific Gram-negative pathogens, the authors of large-scale prevalence studies did not clearly outline the distribution of these types of infections. More inclusive nationwide studies in which both hospital- and community-acquired Gram-negative infections are examined would be beneficial.

## INTRODUCTION

Gram-negative bacteria represent a significant cause of morbidity and mortality in both hospital and community settings worldwide. These pathogens pose a serious threat because their inherent resistance to various environmental conditions and antibiotic treatments can complicate infection management, making them challenging to treat.^1^^,^^2^ Pathogenic Gram-negative bacteria are remarkably adaptable, able to colonize diverse anatomical sites within the human body, and cause a wide range of severe infections, including bloodstream infections (BSIs) and pneumonia.^2^^,^^3^ With the prevalence of antibiotic-resistant Gram-negative bacteria increasing globally, the associated public health burden necessitates new therapeutic interventions and effective infection control strategies. In addition, the ability of these bacteria to acquire and disseminate antibiotic-resistance genes poses a considerable threat to global health, requiring concerted efforts in surveillance, research, and policymaking to mitigate their impact.^1^^,^^2^ As the international medical community deals with the complexities of Gram-negative bacterial infections, understanding current trends and developing new approaches will continue to be crucial for addressing this ongoing issue.

Antibiotic health literacy and regulations governing access and hospital use are less well controlled in Thailand and other countries than they are in the United States. Certain antibiotics are available for purchase in places such as grocery stores.^4^ Patient use without appropriate knowledge and oversight can be risky, not only because it increases the likelihood of resistance but also because these drugs can have dangerous side effects. A 2014 survey of 19,468 Thai residents revealed that half of those who experienced colds, diarrhea, or simple wounds used antibiotics.^4^ High levels of antibiotic use in livestock for the treatment and prevention of infections have promoted high levels of resistance to some clinically useful agents.^5^ This rise in resistance has led to notable decreases in treatment options and delays in care. It has simultaneously exacerbated the antibiotic resistance problem because patients require a range of broad-spectrum antibiotics and still have poor outcomes.^6^ Hospital-acquired infections (HAIs) are a significant concern because many Gram-negative bacteria that cause these infections have become remarkably drug-resistant. Meanwhile, community-acquired infections (CAIs) are proving to have similar and sometimes higher rates of mortality compared with HAIs.

Research in this area over the last 10 years has primarily been focused on determining the different resistance patterns of these bacteria and developing new drug combinations to combat them. The authors of few studies have documented the prevalence and geographical distribution of the leading causes of Gram-negative bacterial infections in Thailand. Understanding these trends geographically and seasonally could help strengthen public health programs.

## MATERIALS AND METHODS

The present article is a narrative review. It contains no new studies involving human participants or animals and is based on previously conducted studies. PubMed and Embase database searches were performed using the search terms ((Thailand[Title] OR Thai[Title]) AND (“Gram-negative bacterial infections”[MeSH Terms] OR “Gram-negative bacteria”[Title/Abstract] OR “*Acinetobacter* baumannii”[Title/Abstract] OR “*Escherichia coli*”[Title/Abstract] OR “*Pseudomonas aeruginosa*”[Title/Abstract] OR “*Klebsiella pneumoniae*”[Title/Abstract] OR “*Burkholderia pseudomallei*”[Title/Abstract] OR sepsis[Title/Abstract] OR fever[Title/Abstract] OR “hospital-acquired infections”[MeSH Terms] OR “community-acquired infections”[MeSH Terms])) AND (adults[MeSH Terms] OR adults[Title/Abstract]). PubMed and Embase database searches were conducted for English-language articles published between 2010 and 2024 in which specific prevalence data on Gram-negative bacteria in Thailand were reported. Studies involving adult populations (≥18 years), studies in which age was not specified, and studies with age ranges that included both children and adults were included. Articles in which data related to Gram-negative bacterial infections were not reported, studies exclusively focused on pediatric populations (under 18 years only), studies in which out-of-scope species, such as Gram-positive bacteria, were evaluated, studies in which only a single organism was evaluated, non-Thailand-specific studies, non-English language publications, and articles published outside the 2010–2024 timeframe were excluded. Of 147 full-text publications initially identified, only 11 met the final inclusion criteria; additional articles identified through hand searches were also included if they met these criteria.

## RESULTS

**Figure 1.**
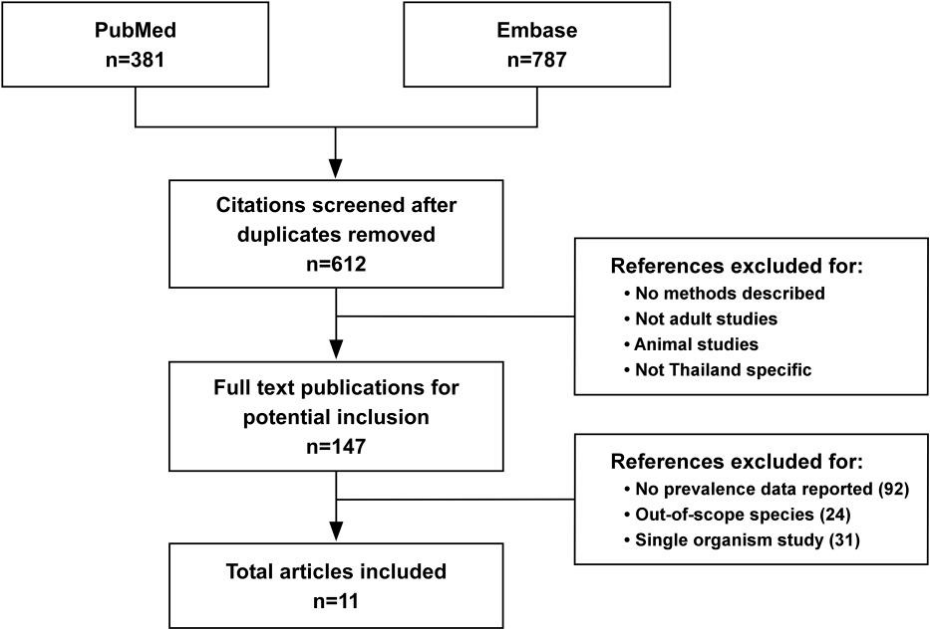
Flowchart summarizing the publications identified via database searches, author-conducted searches, and the selection of studies for the present review.

A summary of the results of the search process used in the present study is shown in Figure 1. The searches yielded 147 full-text publications for potential inclusion, of which only 11 met the final inclusion criteria, which required that the study include specific prevalence data on Gram-negative bacteria in Thailand (Table 1). These papers were focused on key Gram-negative pathogens, including *Acinetobacter baumannii*, *Klebsiella pneumoniae*, *Escherichia coli*, *Pseudomonas aeruginosa*, and *Burkholderia pseudomallei*. *Enterobacteriaceae* and *Salmonella* were also considered in some studies.

**Table 1:** Publications included in the present review identifying the types of Gram-negative bacteria across various settings in Thailand

Location and Publication Year	Hospital Type	Study Years	Percentage of Gram-Negative Bacteria (Out of Total)	Percentages of Each Species of Gram-Negative Bacteria	Age Range	Study Population Description
All provinces in Thailand except Mae Hong Son, Nakorn Nayok, and Bangkok, 2025^7^	35 level A hospitals, 49 level S hospitals, and 27 level M hospitals	January–December 2022	73% of community-onset BSIs and 68% of hospital-onset BSIs	*E. coli* (27% of CAIs and 51% of HAIs), *K. pneumoniae* (23% of CAIs and 55% HAIs), *Acinetobacter* spp. (26% of CAIs and 70% HAIs), *P. aeruginosa* (18% of CAIs and 41% of HAIs), *B. pseudomallei* (not given), *Salmonella* non-typhi (not given)	Not specified	Retrospective study on inpatient culture confirmed infections using the AMASS tool to deduplicate and merge data. The analysis excluded outpatients and non-public facilities.
All Regions of Thailand, 2021^8^	47 hospitals: 7 universities, 36 government, 3 private, and 1 military	October 2017– January 2019	Studies focused only on Gram-negative bacteria	*E. coli* (34.1%), *K. pneumoniae* (25.6%), *Acinetobacter* spp. (22.7%), *P. aeruginosa* (17.6%)	Not specified	Prospective study on clinical isolates from blood, respiratory tract, urine, and sterile body sites collected from both inpatients and outpatients without stratification by disease onset or history. Repeated isolates from the same patient were excluded.
Nakhon Phanom and Tak Provinces, 2024^9^	12 provincial and district hospitals	April 2017–May 2020	17.66% of acute undifferentiated febrile illness	*E. coli* (71.75%), *K. pneumoniae* (13.45%), *B. pseudomallei* (11.66%), *A. baumannii* 1.79%	2–80 years of age	Prospective observational study enrolling inpatients and outpatients with acute undifferentiated febrile illness (≤7 days). Patients with both bacterial and nonbacterial infections were excluded.
Bangkok, 2023^10^	Tertiary care university hospital	October 2016–March 2018	21.7% of patients who had mechanical ventilation	MDR *A. baumannii* (90.8%), MDR *K. pneumoniae* (4.6%), MDR *P. aeruginosa* (4.3%)	>18 years of age	Retrospective observational study of hospitalized adult patients who received continuous mechanical ventilation for ≥48 hours.
Bangkok, 2014^11^	1 university hospital	February– May 2012	70.2% of HAIs	MDR *A. baumannii* (88.7%), ESBL-producing *Enterobacteriaceae* (37.8%), Carbapenem-resistant *P. aeruginosa* (39.3%)	>18 years of age	Cross-sectional study of adult hospitalized patients with positive cultures.
All regions of Thailand, 2017^12^	50 hospitals: 48 government and 2 private	January 2014	36.3% of HAIs	*Acinetobacter* spp. (17.3%), *P. aeruginosa* (9.6%), *Klebsiella* spp. (9.4%)	Not specified	Prospective study of hospitalized patients to assess HAIs.
Ubon Ratchathani Province, 2018^13^	1 tertiary care hospital	March 2013–January 2017	9.9% of the 4,989 patients hospitalized with CAIs	*E. coli* (4.2%), *B. pseudomallei* (3.0%), *K. pneumoniae* (0.8%), *Pseudomonas* spp. (0.7%), *Acinetobacter* (0.6%)	>18	Prospective observational study of hospitalized inpatients with CAIs and systemic manifestations within 24 hours of admission.
Bangkok, 2018^14^	1 university hospital	January 2008–December 2012	Studies focused only on Gram-negative infections	*A. baumannii* (44.79% of AMR infections and 14.96% of non-AMR infections), *E. coli* (22.90% of AMR infections and 21.65% of non-AMR infections), *K. pneumoniae* (14.93% of AMR infections and 18.90% of non-AMR infections), *P. aeruginosa* (5.93% of AMR infections and 43.31% of non-AMR infections)	>18	Retrospective cohort study of hospitalized patients with nosocomial infections acquired in critical care units.
Nakhon Phanom and Sa Kaeo Provinces, 2019^15^	Two provincial hospitals (225–327 beds) and 18 peripheral district hospitals (10–140 beds)	2007–2014 (unspecified months)	73% of community-acquired BSIs and 68% of hospital-acquired BSIs	*E. coli* (27% of CAIs and 51% of HAIs), *Acinetobacter* spp. (26% of CAIs and 55% of HAIs), *K. pneumoniae* (23% of CAIs and 55% of HAIs), *B. pseudomallei* (Not given), *Salmonella* non-typhi (not given), *P. aeruginosa* (Not given)	Not specified	Population-based surveillance of hospitalized patients with clinically indicated blood cultures performed.
Northeast Thailand, 2013^16^	10 provincial hospitals	January 2004–December2010	71.1% of CAIs	*E. coli* (23.1%), *B. pseudomallei (*19.3%)	Not specified	Multicenter surveillance study of hospitalized patients with pathogenic organisms isolated from blood taken within 2 days of hospital admission and without a previous inpatient episode in the preceding 30 days.
Bangkok, 2024^17^	Tertiary university hospital	January 2002–December 2016	57.3% of hospital-acquired bacterial meningitis, 19.1% of community-acquired bacterial meningitis	*A. baumannii* (18.7%), *K. pneumoniae* (16.0%), *E. coli (*8.0%), *P. aeruginosa* (6.7%)	>18 years of age	Retrospective study of hospitalized adult inpatients diagnosed with acute bacterial meningitis.

*A. baumannii* = *Acinetobacter baumannii*; AMR = antimicrobial-resistant; *B. pseudomallei* = *Burkholderia psuedomallei*; BSI = bloodstream infection; CAI = community-acquired infection; *E. coli* = *Escherichia coli*; ESBL = extended-spectrum beta-lactamase; HAI = hospital-acquired information; *K. pneumoniae* = *Klebsiella pneumoniae*; MDR = multidrug resistant; *P. aeruginosa* = *Pseudomonas aeruginosa*.

### The most common Gram-negative bacteria associated with CAIs and HAIs.

*Escherichia coli* and *B. pseudomallei* were most frequently associated with CAIs. *Escherichia coli* is a common cause of urinary tract infections, and *B. pseudomallei* is endemic to Thailand and causes melioidosis. Studies have primarily revealed that *B. pseudomallei* is hyperendemic in the northeastern provinces. A 2007–2014 study on CAIs and HAIs revealed that *E. coli* accounted for 27% of CAIs and 51% of HAIs. *Burkholderia pseudomallei* was among the 10 most common pathogens in one study of community-onset BSIs, ranking second after *E. coli* in adults aged 50–64 years and 65+ years.^15^

Multiple studies have highlighted the prevalence of *Acinetobacter* spp., *P. aeruginosa*, *K. pneumoniae*, and *E. coli* as leading pathogens in HAIs. A 2014 study revealed *Acinetobacter* spp. (17.3%) as the most common, whereas more recent data from 2021 has indicated that drug-resistant strains of *E. coli* (34.1%) and *K. pneumoniae* (25.6%) have become increasingly significant.^8^^,^^12^ In a 2022 study, in which three Thai provinces (Mae Hong Son, Nakorn Nayok, and Bangkok) were excluded, CAIs and HAIs were examined.^7^ Researchers determined that Gram-negative bacteria caused 73% of community-acquired BSIs and 68% of hospital-acquired BSIs.^7^ Of these HAIs, *Acinetobacter* spp. (70%) was the most common, followed by *K. pneumoniae* (55%) and *E. coli* (51%). For CAIs, *E. coli* (27%) was slightly more prevalent than *Acinetobacter* spp. (26%) and *K. pneumoniae* (23%). This study did not include the prevalence of *B. pseudomallei* relative to other Gram-negative infections. However, in 2022, 4,407 cases of *B. pseudomallei* infection were reported, with a mortality rate of 27.7%. The incidence of melioidosis increased by 50% based on the number of cases annually compared with the number of cases in 2012–2015.^7^

### Geographic variability.

Analysis of available studies highlights significant regional variation in pathogen distributions and gaps in surveillance coverage for Gram-negative infections across Thailand. Four of 11 included studies were conducted exclusively in Bangkok at tertiary care university hospitals, specifically Siriraj Hospital and Ramathibodi Hospital.^10^^,^^11^^,^^14^^,^^17^ These larger urban hospital studies are primarily focused on multidrug-resistant (MDR) pathogens and HAIs. In contrast, provincial and rural hospitals, which have a broader range of capacities and patient populations, conduct more studies on CAIs.^9^^,^^15^^,^^16^

There are clear geographic patterns for specific pathogens, most notably *B. pseudomallei*, which consistently appears to be more endemic in the northeastern provinces. A study of community-acquired bacteremia in Northeast Thailand conducted from 2004 to 2010 revealed *B. pseudomallei* as the second most common pathogen (19.3%) after *E. coli* (23.1%).^16^ Within the northeastern provinces, Nakhon Phanom recorded the highest community-acquired bacteremia rate at 57.8 per 100,000 people compared with 18.4 in Loei Province.^16^ Geographic surveillance gaps are worsened by variations in diagnostic readiness. Many areas of Thailand are not considered endemic for *B. pseudomallei*, leaving hospitals unprepared to test and treat melioidosis.^18^ This can contribute to underdiagnosis and delayed treatment, which could explain some of the differences in regional mortality reports.

There were also significant variations among provinces in terms of *E. coli* levels. A 3-year observational study revealed that 16.5% of acute undifferentiated fever cases in Nakhon Phanom were caused by *E. coli*, compared with 7.6% in Tak Province.^9^ However, this study did not include any analysis of the underlying factors that might lead to this difference. *Klebsiella pneumoniae* also exhibited significant geographic variability, representing 13.45% of Gram-negative causes of undifferentiated fever in a northeastern study and 25.6% of critically drug-resistant Gram-negative bacteria in a national hospital surveillance study.^8^^,^^9^ Although these studies reveal geographic variation amongst various pathogens, direct comparisons are limited by fundamental differences in study design and objectives.

There is a significant knowledge gap regarding infection patterns in rural Thailand, with a direct correlation to the more limited healthcare infrastructure, which impacts diagnostic capabilities and reporting accuracy. Provincial and rural hospitals frequently lack the laboratory equipment necessary to complete comprehensive reports for the National Notifiable Disease Surveillance System. Despite Thailand having 77 provinces, the largest national study explicitly excluded three provinces (Mae Hong Son, Nakorn Nayok, and Bangkok); minimal data are available for the Southern and Northern regions.^7^

### Leading Gram-negative infections that cause bacteremia and undifferentiated fever.

The authors of a 2004–2010 study observed CAIs and reported that the leading causes were *E. coli* (23.1%) and *B. pseudomallei* (19.3%), with the incidence rate of community-acquired bacteremia in Northeast Thailand increasing from 16.7 to 38.2 per 100,000 people per year between 2004 and 2010.^16^ Nakhon Phanom Province had the highest rate of community-acquired bacteremia, with 57.8 per 100,000 people in 2010, whereas Loei Province had an incidence of 18.4 per 100,000 people.^16^ Of the deaths that occurred due to community-acquired bacteremia in this study, 59.1% occurred during the first 2 days of admission.^16^ The authors of a 3-year observational study conducted from 2017 to 2020 in Nakhon Phanom and Sa Kaeo Provinces examined the causes of acute undifferentiated fever and concluded that 17.66% of these cases were due to Gram-negative bacteria.^9^ *Escherichia coli* caused 71.75% of cases, whereas *K. pneumoniae* caused 13.45%, *B. pseudomallei* caused 11.66%, and *A. baumannii* caused 1.79%.^9^ The prevalence of each Gram-negative bacterial species differed significantly between the two provinces; for example, *E. coli* was found in 16.5% of patients in Nakhon Phanom Province but only 7.6% in Tak Province.^9^

### Antimicrobial-resistant and MDR Gram-negative bacterial infections.

Four of the studies were conducted in Bangkok, and all but one were conducted at Siriraj Hospital, a tertiary care university hospital.^10^^,^^11^^,^^14^^,^^17^ The one study that took place outside of Siriraj Hospital was conducted at Ramathibodi Hospital, which has several critical care units that serve Bangkok and the surrounding areas. The authors of this study collected data from 2008 to 2012 and explicitly focused on Gram-negative infections to determine which pathogens were the leading antimicrobial-resistant (AMR) microorganisms versus non-antimicrobial resistance-causing microorganisms.^14^ Of the AMR infections, the leading causes were *A. baumannii* (44.79%), *E. coli* (22.90%), *K. pneumoniae* (14.93%), and *P. aeruginosa* (5.93%). Of the non-AMR infections, the leading causes were *P. aeruginosa* (43.31%), *K. pneumoniae* (18.90%), *E. coli* (21.65%), and *A. baumannii* (14.96%).^14^ In this study, it was estimated that if AMR bacteria were eliminated, there would be 48,258 fewer deaths in Thailand each year.^14^

Similarly, the authors of a separate study conducted in Bangkok examined MDR Gram-negative bacterial infections among patients receiving mechanical ventilation.^10^ Of the cases of ventilator-associated tracheobronchitis and pneumonia, 21.7% were caused by Gram-negative bacteria. This study revealed that MDR *A. baumannii* caused 90.8% of these infections, whereas 4.6% were caused by MDR *K. pneumoniae*, and 4.3% were caused by MDR *P. aeruginosa*.^10^

An additional report on HAIs conducted between 2002 and 2016 revealed that 57.3% of hospital-acquired and 19.1% of community-acquired bacterial meningitis cases were caused by Gram-negative bacteria.^17^ The causes of these infections were *A. baumannii* (18.7%), *K. pneumoniae* (16.0%), *E. coli (*8.0%), and *P. aeruginosa* (6.7%).^17^ Lastly, in 2014, in Bangkok, a study was conducted to determine the causes of HAIs and revealed that 70.2% of HAIs were due to Gram-negative bacteria, with an overall MDR prevalence of 48.8% among Gram-negative isolates. Among the specific MDR pathogens, MDR *A. baumannii* exhibited the highest resistance rate at 88.7%, followed by carbapenem-resistant *P. aeruginosa* at 39.3% and extended-spectrum beta-lactamase-producing *Enterobacteriaceae* at 37.8%.^11^ This was the only study that included *Enterobacteriaceae*.

## DISCUSSION

The findings of this review highlight the significant burden of Gram-negative bacterial infections in both hospital-acquired and community-acquired settings across Thailand. Hospital-acquired infections are more common than CAIs, according to the available studies.^11^ Interestingly, the identification of a causative agent is more likely to be determined in CAIs. In a study focused on bacterial meningitis in Thailand, causative pathogens were identified ∼20% more frequently in community-acquired cases than in HAIs (62.1% versus 41.9%).^17^ However, this study also indicated that the identification rate of organisms, both for HAIs and CAIs, is only determined approximately half of the time.^17^ The authors of very few studies in the present review evaluated Gram-negative bacteria in the context of both HAIs and CAIs.

*Escherichia coli* and *B. pseudomallei* were the predominant pathogens in CAIs, whereas *A. baumannii*, *K. pneumoniae*, and *P. aeruginosa* were the most common pathogens in HAIs. In Thailand, underreporting certain emerging bacteria, such as *B. pseudomallei*, is a significant concern because it hampers disease burden assessment and impacts public health efforts. *Burkholderia pseudomallei* causes melioidosis and is generally considered a CAI due to environmental exposure to contaminated soil or water.^19^ Melioidosis manifests as a range of diseases, from chronic localized infections to acute pneumonia and life-threatening sepsis.^20^ Death from melioidosis frequently occurs because of uncontrolled bacteremia.^21^ Recent findings have revealed the need for extra caution surrounding this organism outside of its traditional environmentally acquired route of infection. In 2021, 25 patients were infected with *B. pseudomallei* through contaminated tap water while being treated at a coronavirus disease 2019 field hospital in Saraburi Province in Central Thailand.^19^ Generally, recommendations for melioidosis prevention do not include tap water disinfection; however, the authors of this study emphasized the need for enhanced awareness of this risk.^19^ The increase in the prevalence of melioidosis may be due in part to an increase in diabetes, a significant risk factor for the disease, as well as improved diagnostic tools.^7^ A significant increase in deaths from melioidosis was reported in 2022 because of a stewardship program that improved reporting among referral hospitals.^7^ Many parts of Thailand are not considered endemic for *B. pseudomallei*, leaving hospitals and providers unprepared to test and treat this disease.^18^ Enhancing efforts to map the prevalence and precise geographic distribution of this emerging bacterial pathogen will be necessary to develop targeted public health interventions and improve health outcomes.

Despite research revealing that bacterial infections are reaching critical numbers globally, especially AMR strains, these infections continue to rise. One of the main concerns expressed in hospitals using the National Notifiable Disease Surveillance System (NNDSS) is that these systems are labor-intensive and often require manual calling or the submission of documentation on reportable diseases.^22^ In particular, primary hospitals frequently lack laboratory equipment to complete these reports, resulting in incomplete submissions that often do not include culture results and are therefore inaccurate. For example, despite melioidosis being a notifiable disease that has been widely documented to cause significant morbidity and mortality across Thailand, only 10 cases of death due to *B. pseudomallei* infections are reported each year.^22^ Having detailed and accurate numbers could provide valuable insight into where funding and resource allocation would yield the greatest benefit. Ideally, there should be an accessible way to track infectious disease cases nationally. In 2021, a new tool called AMASSplus (AMASS Corporation, Bangkok, Thailand) was trialed in six referral hospitals across Thailand, which enabled the successful recording and uploading of cases of notifiable diseases.^22^ The results of the melioidosis tracking differences, with two deaths reported via the NNDSS and 134 reported via AMASSplus, were sent to the Department of Disease Control in Thailand. This tool is now being used to supplement the NNDSS for more accurate tracking moving forward.^22^

The WHO includes carbapenem-resistant *A. baumannii* (CRAB) on its priority pathogens list as a critical priority for antimicrobial research and development because of its resistance to current treatments and high mortality rate.^23^ The prevalence of MDR organisms in HAIs, especially CRAB, poses a significant challenge to treatment because limited therapeutic options are available. Multiple studies across Thailand have revealed that isolates of *A. baumannii* have spread and are resistant to seven antibiotic classes, including penicillin, β-lactam/β-lactamase inhibitor combinations, cephalosporins, carbapenems, aminoglycosides, fluoroquinolones, and folate pathway antagonists. In a study in which excess deaths due to antibiotic resistance were examined, CRAB was determined to cause the highest number of deaths.^18^ Carbapenem-resistant *A. baumannii* is particularly problematic in intensive care unit settings, where patients are already at increased risk of infection and have weakened immune systems.^18^ In a study at Siriraj Hospital, *A. baumannii* was identified as the most prevalent pathogen causing ventilator-associated tracheobronchitis and ventilator-associated pneumonia.^10^

The WHO assigned the highest priority to carbapenem-resistant Enterobacteriaceae. These highly resistant bacteria have limited treatment options, often necessitating the use of colistin, a last-resort drug with high nephrotoxicity and other potential side effects. Unfortunately, colistin is beginning to lose its effectiveness, and a drastic increase in its use in both animals and humans has led to the emergence of resistant strains.^24^ A recent study conducted between 2017 and 2021 revealed that tigecycline resistance in *K. pneumoniae* has also been increasing tremendously.^25^ Tigecycline is one of the last-resort antibiotics for treating infections caused by *K. pneumoniae* strains that are both carbapenem- and colistin-resistant.^25^ From 2017 to 2021, 91.7% of the *K. pneumoniae* isolates in this study were tigecycline-resistant.^25^ The high levels of antibiotic resistance and the increasing frequency of the exchange of genes between animal pathogens and human pathogens highlight the need for a One Health or holistic approach when developing solutions.^26^^–^^28^

Therapeutic options for MDR bacteria are limited, making the selection of clinical treatments extremely important. In Thailand, antibiotics remain widely accessible and are sometimes used without culture tests or confirmed diagnoses. Enhancing education on proper antibiotic use and continuing to bolster regulations governing their use would help address these issues.^29^ Local epidemiological studies offer some insights into antibiotic usage throughout Thailand.^30^ Research conducted between 2018 and 2020 revealed that after adjusting for confounders, 106 of 1,385 cases (7.7% of deaths) were caused by antibiotic-resistant pathogens.^18^ A study conducted from 2017 to 2020 on acute undifferentiated febrile illnesses revealed that 10% of patients who received antibiotics had a disease with a non-bacterial cause.^9^ These observations highlight the need for increased training and support for proper antibiotic use. Conducting comprehensive national studies across both hospital and community settings would help address these gaps and provide accurate data for public health interventions. These findings could be used to advocate for improved infection control practices, enhanced antimicrobial stewardship, and more regional surveillance to combat the rising threat of MDR Gram-negative infections in Thailand.

### Limitations.

The present review has several important methodological and interpretational limitations. First, the findings are based on only 11 included studies, which limits the generalizability of the conclusions. The studies varied significantly in design, patient populations, and data reporting, introducing heterogeneity that complicates comparisons. Second, laboratory testing limitations may have impacted accuracy because variability in sensitivity and specificity of bacterial culture methods across hospitals can lead to misclassification or underdiagnosis of infections. Third, potential bias arises from differences across study sites and time periods, with most studies conducted at large tertiary care centers in Bangkok, while rural and provincial hospitals were underrepresented.

Additionally, surveillance gaps and inconsistent diagnostic capacity, particularly in resource-limited settings, likely contributed to underreporting of pathogens. Excluding pediatric populations further limits the applicability of these findings to all age groups. Finally, methodological and reporting inconsistencies, such as variations in case definitions, outcome measures, and pathogen categorization, further limit study comparability. These limitations underscore the need for standardized laboratory practices, expanded national surveillance, and large-scale, multicenter studies to better characterize the epidemiology of Gram-negative infections in Thailand.

## CONCLUSION

The ever-growing prevalence of Gram-negative bacterial infections acquired from hospitals and community settings in Thailand is a significant public health concern. The key pathogens causing these infections, including *A. baumannii*, *K. pneumoniae*, *E. coli*, *P. aeruginosa*, and *B. pseudomallei*, are not only becoming more numerous but, in many cases, are also becoming more resistant to available medications. Although there is a wealth of studies and continuing research into antibiotic resistance patterns and treatments, there remains a critical lack of data on the prevalence and geographic distribution of these infections in Thailand. The current review highlights opportunities for improvement in nationwide surveillance, particularly for underreported pathogens such as *B. pseudomallei*. These data are essential for developing tailored and effective public health programs. The lack of comparability among data sources particularly leads to difficulties in understanding trends and making informed decisions at a policy level.^31^ Pathogen-specific data can aid in tracking the occurrence of epidemiologically important pathogens and newly emerging pathogens as they arise.^31^

Addressing the issue of increasing antibiotic resistance will require a multifaceted approach, and expanded regulations related to antibiotic availability and enhanced, easier-to-use reporting systems will likely be beneficial. Creating and enforcing new laws governing the distribution and use of antibiotics worldwide may ultimately be needed to reduce the selection pressure for drug-resistant bacteria. Policymakers and researchers should continue advocating for evidence-based guidelines and antimicrobial stewardship programs while improving public awareness and education. Public health officials can more easily address human, animal, and environmental factors contributing to this growing problem by increasing the surveillance of these bacteria and antibiotic use. Ultimately, more large-scale studies are needed to better understand the impact of Gram-negative infections in Thailand and the actual morbidity and mortality associated with disease and death caused by this important group of pathogens.
